# Challenges to Adolescent HPV Vaccination and Implementation of Evidence-Based Interventions to Promote Vaccine Uptake During the COVID-19 Pandemic: “HPV Is Probably Not at the Top of Our List”

**DOI:** 10.5888/pcd19.210378

**Published:** 2022-03-31

**Authors:** Grace Ryan, Paul A. Gilbert, Sato Ashida, Mary E. Charlton, Aaron Scherer, Natoshia M. Askelson

**Affiliations:** 1 College of Public Health, University of Iowa, Iowa City, Iowa; 2 Now with University of Massachusetts Chan Medical School, Worcester, Massachusetts; 3 Carver College of Medicine, University of Iowa, Iowa City, Iowa

## Abstract

**Introduction:**

The COVID-19 pandemic has prevented many adolescents from receiving their vaccines, including the human papillomavirus (HPV) vaccine, on time. However, little is known about the impact of the pandemic on implementation of clinic-level evidence-based interventions (EBIs) that help to improve HPV vaccine uptake. In this qualitative study, we explored the pandemic’s impact on EBI implementation and HPV vaccine delivery.

**Methods:**

During August–November 2020, we interviewed clinic managers in a rural, midwestern state about their experiences implementing EBIs for HPV vaccination during the COVID-19 pandemic. We used a multipronged sampling approach with both stratified and purposive sampling to recruit participants from Vaccines for Children clinics. We then conducted a thematic analysis of transcripts.

**Results:**

In interviews (N = 18), 2 primary themes emerged: decreased opportunities for HPV vaccination and disruption to HPV-related implementation work. Most participants reported decreases in opportunities to vaccinate caused by structural changes in how they delivered care (eg, switched to telehealth visits) and patient fear of exposure to COVID-19. Disruptions to EBI implementation were primarily due to logistical challenges (eg, decreases in staffing) and shifting priorities.

**Conclusion:**

During the pandemic, clinics struggled to provide routine care, and as a result, many adolescents missed HPV vaccinations. To ensure these adolescents do not fall behind on this vaccine series, providers and researchers will need to recommit to EBI implementation and use existing strategies to promote vaccination. In the long term, improvements are needed to make EBI implementation more resilient to ensure that progress does not come to a halt in future pandemic events.

**Summary**

**What is already known on this topic?**

COVID-19 has affected health care delivery, including delivery of human papillomavirus (HPV) vaccine; however, little is known about the impact of the pandemic on implementation efforts related to promoting HPV vaccination.

**What is added by this report?**

We explored challenges to HPV vaccination through the perspectives of clinic managers in family practice and pediatric clinics and found that many efforts to implement evidence-based interventions to promote HPV vaccination have similarly been disrupted by the pandemic.

**What are the implications for public health practice?**

Without a recommitment to implementation by researchers and practitioners alike, we risk leaving many adolescents unprotected against HPV.

**MEDSCAPE CME**

In support of improving patient care, this activity has been planned and implemented by Medscape, LLC and *Preventing Chronic Disease*. Medscape, LLC is jointly accredited by the Accreditation Council for Continuing Medical Education (ACCME), the Accreditation Council for Pharmacy Education (ACPE), and the American Nurses Credentialing Center (ANCC), to provide continuing education for the healthcare team.

Medscape, LLC designates this Journal-based CME activity for a maximum of 1.00 AMA PRA Category 1 Credit(s)™. Physicians should claim only the credit commensurate with the extent of their participation in the activity.

Successful completion of this CME activity, which includes participation in the evaluation component, enables the participant to earn up to 1.0 MOC points in the American Board of Internal Medicine’s (ABIM) Maintenance of Certification (MOC) program. Participants will earn MOC points equivalent to the amount of CME credits claimed for the activity. It is the CME activity provider’s responsibility to submit participant completion information to ACCME for the purpose of granting ABIM MOC credit.

**Release date:** March 31, 2022; **Expiration date:** March 31, 2023

**Learning Objectives**

Upon completion of this activity, participants will be able to:

1. Analyze the application of human papillomavirus vaccine in the US before and during the COVID-19 pandemic
2. Distinguish major challenges to human papillomavirus vaccination during the pandemic
3. Analyze patient-related variables associated with rates of human papillomavirus vaccination during the COVID-19 pandemic
4. Identify practice changes likely to continue after the COVID-19 pandemic

**EDITOR**

Ellen Taratus

Senior Editor

Preventing Chronic Disease

Disclosure: Ellen Taratus has disclosed no relevant financial relationships.

**CME AUTHOR**

Charles P. Vega, MD

Health Sciences Clinical Professor of Family Medicine

University of California, Irvine School of Medicine

Irvine, California

Disclosure: Charles P. Vega, MD, has disclosed the following relevant financial relationships:

Served as an advisor or consultant for: GlaxoSmithKline; Johnson & Johnson

**AUTHORS**

Grace Ryan, PhD, MPH

College of Public Health, University of Iowa, Iowa City, Iowa

Paul A. Gilbert, PhD

College of Public Health, University of Iowa, Iowa City, Iowa

Sato Ashida, PhD

College of Public Health, University of Iowa

Mary E. Charlton, PhD

College of Public Health, University of Iowa, Iowa City, Iowa

Aaron Scherer, PhD

Carver College of Medicine, University of Iowa, Iowa City, Iowa

Natoshia M. Askelson, PhD, MPH

College of Public Health, University of Iowa, Iowa City, Iowa

## Introduction

Since March 2020, the pandemic caused by SARS-CoV-2 has affected nearly all aspects of daily life, particularly in the ways in which health care is provided. Because of fear of coming into clinics and the preference for telehealth appointments, one area that has been especially affected is pediatric and adolescent immunization (1,2). Before the pandemic, data from the 2019 National Immunization Survey–Teen showed that only 54% of US adolescents aged 13 to 17 were up to date with the human papillomavirus (HPV) vaccine series (3). An analysis to estimate the impact of the pandemic on HPV vaccination found that vaccination rates were 75% lower during the pandemic compared with prior periods, and statistical modeling showed that these lower rates could lead to increases in the incidence of genital warts, cervical intraepithelial neoplasia, and other HPV-related cancers if adolescents do not catch up on the HPV vaccine (4). Adolescence is a critical period for HPV prevention. The Advisory Committee for Immunization Practices recommends administration of 2 doses of HPV vaccine for children and adolescents aged 9 to 14 years (5). Initiating administration early in adolescence is linked to higher rates of on-time completion (6) and increased effectiveness (7,8). Therefore, understanding the extent to which the pandemic has affected adolescents’ ability to get vaccinated is imperative.

Challenges in vaccinating adolescents during the last decade have led researchers and quality improvement (QI) staff to develop evidence-based interventions (EBIs) and strategies to assist clinical staff in increasing HPV vaccination. For example, commonly used EBIs include reminder/recall systems, standing orders for HPV vaccination, and provider assessment and feedback (9,10). Because of the complexity of these EBIs, a substantial amount of work happens “behind the scenes” in clinics. These implementation efforts to increase HPV vaccination rates (9–11) are often conducted independently or in collaboration with academic and community partners and led by administrative, non–patient-facing staff.

Nearly 2 years into the pandemic, little is known about the impact of the pandemic on implementation of these EBIs to encourage vaccinations or about the experience of clinics that continued to provide routine care during the pandemic. Our attention now needs to turn to these lesser studied impacts on clinic practices that have implications for future health outcomes of adolescents. The aim of this qualitative study was to explore the experiences of clinics that continued to provide routine care during the pandemic and the impact of the pandemic on ongoing implementation efforts to promote HPV vaccination.

## Methods

This study was part of a larger project using the Consolidated Framework for Implementation Research (CFIR; https://cfirguide.org/) to understand barriers and facilitators to EBI implementation focused on HPV vaccination in clinics integrated or affiliated with large health care systems in Iowa. However, only results related to the impact of COVID-19 are reported here. We conducted semistructured interviews with clinic managers or administrators working in Vaccines for Children (VFC) clinics in large health care systems in Iowa from August through November 2020. The University of Iowa Review Board determined that this study did not meet the criteria for human subjects research. All participants were provided with information about the study and its purpose, compensation, the voluntary nature of their participation, and the researcher’s contact information. We offered a $25 gift card to all participants to thank them for their time.

We used multipronged sampling that included stratified sampling of VFC clinics in Iowa. First, researchers examined the list of VFC clinics in the state (N = 594) and excluded clinics that were either not pediatric/family practice clinics or not integrated or affiliated with a larger health care system, resulting in a final list of 305 clinics that met inclusion criteria. We stratified clinics by congressional district and rurality; a random sample of clinics (n = 5) was drawn from each stratum (n = 8); we repeated this process, ultimately recruiting from a sample of 80 clinics and completing 9 interviews. Up to 6 attempts were made to contact the clinic manager at each clinic, either by email or telephone. Common reasons for refusal were lack of time due to COVID-19 or not currently having a staff member in an administrative or management position. When this approach did not achieve thematic saturation in interviews, directed recruitment efforts were made through professional networks. Throughout the interview process transcripts were reviewed for thematic saturation, and recruitment ended when it was determined saturation had been reached.

We adapted the interview guide from the CFIR (12). In addition to questions to address the CFIR constructs, we included questions to address the impact of COVID-19 on HPV vaccination delivery and on implementation of EBIs for HPV vaccination. Before the interview, we sent all participants a brief survey to collect demographic information about them and their clinic. All interviews were conducted by the first author (G.R.) via telephone and audio recorded. A third-party service was used for verbatim transcription.

We generated frequencies and appropriate descriptive statistics for survey items capturing information on participant and clinic characteristics. To analyze the interviews, we conducted thematic analysis (13) that explored the impact of COVID-19. In the first round of coding, we used Nvivo version 12 (QSR International) to code transcripts and created a code to identify information on the pandemic (“impact of COVID-19”). This process was completed by the first author and a trained student in a master of public health program. We then analyzed the data coded under “impact of COVID-19” using a thematic analysis approach (13) to identify themes and subthemes.

## Results

We completed interviews with 18 individuals; interviews ranged from 19 to 50 minutes, averaging 32 minutes. Of the 18 participants, 8 were aged 27 to 39, all were women, 14 worked in a rural clinic, and 12 worked in general practice or family medicine clinics (Table 1).

**Table 1:** Characteristics of Clinic Managers and Administrators (N = 18) and Clinics Participating in Semistructured Interviews on Adolescent HPV Vaccination During the COVID-19 Pandemic, Iowa, August–November 2020

Characteristic	Number
**Age, y^a^**	
18–26	1
27–39	8
40–59	7
≥60	1
**Time employed at current clinic, y**	
1–3	6
4–6	4
≥6	8
**Sex**	
Male	0
Female	18
**Rurality of clinic**	
Rural	14
Urban	4
**Clinic specialty^b^**	
Pediatrics	6
General practice or family medicine	12
Gynecology	1

a One participant did not report age.

b Participants could select ≥1 clinic specialty.

Two primary themes emerged in all interviews under the parent code of impact of COVID-19: decrease in HPV vaccination and routine care and impact of the pandemic on implementation work (Table 2). In a minority of interviews, a third theme was also identified: patient safety improvements.

**Table 2:** Themes, Subthemes, and Representative Quotes Resulting From Semistructured Interviews on Adolescent HPV Vaccination During the COVID-19 Pandemic, Iowa, August–November 2020

Theme/subtheme	Representative quotes
Theme: Decreased opportunities for HPV vaccination and routine care	
Structural barriers	[Interviewer: Are there any complications with any of this work that have come up during COVID that’s affected your ability to do any of it?] Participant: Probably the only thing would be that back a few months ago, we all had to cut hours because of location status. But now we’re all back pretty much up to our full-time status. Just got behind during the months of pretty much April, May, and June. (I-8)
	I mean, there’s a lot of infection control, a lot more infection control focus with our masking mandate and [having patients who are virtual]. Virtual visits are available now. The virtual waiting room to have patients, you know, waiting in their car and communicating via secure text. So, a lot of technological advances were made and pushed through because of this. So, a lot of workflow got changed. (I-9)
	So, we have talked as a whole if all of that needs to be looked at just because we had for the safety of patients pushed out appointments if necessary, or we try to do them virtually if possible, region-wide for everybody, not just pediatrics. And I think what came back for most of us as administration is, yeah, we recognize that yes some of our metrics could have been missed due to those time periods. (I-1)
Patient/parental fear of coming into clinic	But like I said, the numbers are down. Parents are just very leery of bringing them in to the doctor’s office. They’ve increased a little bit, the last month or so. (I-4)
	I mean, our patients were less likely to come in, we had a lot of very fearful families. And even still, they still don’t want to bring their children into an area where they’re going to encounter other people. (I-3)
	So just parents that are more worried about coming into the office, just trying to reassure them that we’re keeping kids away from them. (I-18)
Theme: Disruption to ongoing EBI implementation work	
Logistical challenges	[Interviewer: And are you still seeing that QI person even over the last six months with COVID?] Participant: No, they were furloughed, but they are back for the last two months I think, and back with a vengeance. They’re working hard to get us back where we need to be. (I-6)
	[Interviewer: And in terms of your HPV vaccination work, have any new complications arisen during COVID-19? Were you able to go out to the schools to do a similar sports physical push this year, things like that?] We were not because schools suspended in March and the orientation usually occurred end of March, early part of April . . . so school wasn’t in session at that time. So we missed that opportunity. (I-6)
Shift in priorities	I think prior to COVID happening, we were putting plans in place on how to increase immunizations, whether that’s signage in the rooms and just communicating with patients and parents about why this is beneficial for your child or for yourself. And then obviously COVID, and that kind of just threw everything quality improvement out the window, while you’re trying to focus on, how the heck are we going to do this? (I-2)
	A lot has changed prior to COVID and now just trying to ensure that we adhere to all the new guidelines and make sure that we have staff, that we protect our staff as well with PPE and all of those things that are constantly changing, as we’re entering into the search plan. I would say that’s probably [leadership’s] main focus right now. (I-15)

Abbreviations: EBI, evidence-based intervention; HPV, human papillomavirus; I, interview; PPE, personal protective equipment; QI, quality improvement.

### Decreased opportunities for HPV vaccination and routine care

Overall reduction in in-person clinic visits due to the pandemic posed tremendous challenges to the clinics in delivering HPV vaccinations. Participants spoke about 2 main challenges in being able to vaccinate patients. First, all clinics had to implement new protocols to safely treat patients, which included reducing overall patient volume and switching to telehealth visits, both of which resulted in fewer adolescents being seen in person. To reduce patient volume, many participants reported that a respiratory clinic was established to physically separate sick patients from well patients, and this meant less time and capacity to see well patients. As one participant described, “[R]ight now, the biggest priority in our clinic is the sick clinic; we’re doing COVID testing in our sick clinic. That’s our biggest area here” (Interview-8). Other participants noted that shifts to virtual visits presented numerous challenges to HPV vaccination. In the first place, these changes required time to implement, and in the second place, specific to HPV vaccination, it meant that “when they’re pushing virtual visits, it’s not going to get them into the door to get those vaccines done” (Interview-11).

The second main challenge was fear of COVID-19 exposure. Most participants reflected on the fear among their patient population of coming into clinics for preventive care such as vaccinations and being exposed to the virus. Although none of the participants had calculated exact numbers for the reduction in vaccinations, nearly all identified patient fear of COVID-19 as a barrier to vaccinating adolescents. As one participant summarized, “[F]or quite a while in the spring, people were really reluctant to come to the doctor’s office, because they felt like we’re a hotbed of disease” (Interview-10). Parents and guardians were not the only ones afraid of attending routine care visits; the clinics themselves shut down for a certain period, and schools made allowances about attendance at sports physicals in recognition that parents may not want to bring their children into clinics. For example, one participant reported that “in April [2020] . . . [the clinic] essentially cancelled every other visit except zero- to five-[year-old patients]” (Interview-3). Another participant spoke about the ruling that schools allowed prior years’ sports physicals to count for school entry, so they “missed some opportunity this year getting in [their] normal amounts” of those visits (Interview-9). Together, the shifts made in clinics to provide patient safety and the hesitancy among parents to take their children into clinics translated into a reduction in HPV vaccinations.

### Disruption to ongoing EBI implementation work

We identified 2 subthemes: disruptions due to logistical challenges and disruptions due to shifts in priorities during the pandemic. All participants noted that the COVID-19 pandemic had a negative impact on their ability to carry out ongoing projects and implementation of EBIs to increase HPV vaccination rates. Several participants spoke about previous efforts with QI teams for HPV vaccination projects. As one clinic manager described, “[I]t’s just kind of fizzled off with . . . COVID” (Interview-14). Others reflected on projects that were ongoing with external partners that had been forced to halt because of the pandemic. For example, one participant spoke about a project with a pharmaceutical company to implement new strategies to promote HPV vaccination that they had started, but with the pandemic “all those meetings and such came to an end because [they] couldn’t meet in person anymore” (Interview-6). Another clinic manager spoke about a school outreach program to promote HPV and other adolescent vaccines in which their clinic usually participates during the spring. However, she reflected that “school wasn’t in session at that time. So, we missed that opportunity” (Interview-5). Finally, several participants noted that because of the pandemic, pharmaceutical representatives who are usually allowed into the clinic to provide education were not able to come and that the regularly scheduled state immunization conference was cancelled. These participants reported that these education opportunities are the primary way staff and providers learn about updates to HPV vaccination and best practices and motivate staff to implement EBIs.

These interruptions were due to both logistical challenges and shifts in priorities that were necessitated by the pandemic. For example, early in the pandemic, many participants noted that because of shut-downs in routine care services, non–patient-facing staff were furloughed while multiple providers were out of the office due to mandatory quarantines resulting from COVID-19 exposure or infection. As one participant noted, “When we [were] short-staffed . . . that changed a lot of different workflows” (Interview-2), which meant increased time was spent to create new workflows, detracting from time available for other projects. In addition to creating staff shortages, the pandemic also disrupted regular communication between participants and others working on EBI implementation or HPV vaccine promotion. For example, one participant noted that their QI team had been redirected to focus on COVID and so “prior to COVID [they] were meeting once a month. But since COVID, [they’ve] gotten pulled into more of the COVID-related areas” (Interview-8). Another clinic manager noted that all her team communication had to switch to an online format and “there are some challenges of not having that person in front of you to talk to” (Interview-5) and that this lack of in-person communication had a particularly negative impact on implementation work. Related to fears of COVID-19 transmission, one participant noted that the clinic had to pull all educational materials from waiting areas because it didn’t “want to lay them out for the patient to pick up” (Interview-4).

Beyond these logistical challenges, the inability to work on EBI implementation and projects related to HPV vaccination was primarily due to the shift in priorities. As one participant summarized it, “[R]ight now with COVID, I would be amiss if I didn’t say HPV [is] probably not at the top of our list. We’re trying to make sure that people are staying healthy” (Interview-17). Many participants spoke about the challenges of maintaining safety protocols and the extra work that came along with that, and how these challenges had led to a reduced focus on HPV vaccination overall. Another participant noted that these shifts were not just occurring in clinics but also that “COVID as a whole has changed our health organization. Looking at how do we bring people in safely has been a huge thing” (Interview-1). This need to focus on patient safety, above all else, has been necessary, but all participants who spoke about these shifts said that it meant they have not had time to focus on EBI implementation for HPV vaccination.

### Patient safety improvements for infection control made during the COVID-19 pandemic

Finally, in discussing implementation of EBIs for HPV vaccination, several participants spoke more generally about how the pandemic has changed health care delivery and lessons learned for the future. These participants spoke about some positive changes that have resulted from the need to be more creative about health care delivery and patient safety. They reflected that there are likely to be some permanent changes to health care delivery for their clinics and health care systems that may have implications for how EBIs are implemented. Several reported that the health care systems their clinics are affiliated with had created special respiratory clinics ― designed to control infection ― to care for COVID-19 patients. One reported that “we plan on keeping the respiratory clinic going forever. With all our respiratory stuff, it just makes sense, really” (Interview-16). Another common change was creating separate entrances or times for well and sick patients to be seen. One participant said they “had to reinvent the wheel as far as what keeps people safe, and how [they] still operate and get things that keep people healthy, without giving them the opportunity to catch something” (Interview-1). Because of the attention they devoted to these efforts, this participant spoke with her team about continuing with these changes throughout respiratory syncytial virus (RSV) season and indicated that many of these changes make “the most sense to keep the most majority of the people healthy coming in” (Interview-1). Implications of these kinds of permanent changes are not yet known, although one participant noted that in her clinic this change had resulted in fewer staff members being available for routine preventive care in the short term.

## Discussion

Results from these interviews highlight a unique perspective on the impact of the COVID-19 pandemic on adolescent HPV vaccination, that of administrative clinic staff, most of whom work in rural areas. Participants in this study, while not directly involved in health care delivery, manage much of the work that happens behind the scenes to ensure patients receive the care they need. Across interviews, the impact of the pandemic on not only adolescent HPV vaccination but on all health care delivery and related EBI implementation work was evident. Interviews focused on implementation of EBIs for HPV vaccination specifically, but many of the barriers reported in relation to this area also applied to other areas. Data from 2020 identified sharp decreases in adolescent and pediatric vaccination (1,2) as well as well-child visits (14) that have likely persisted into 2021. Now, with the authorization of the COVID-19 vaccine for both adolescents and children, HPV vaccination may not be at the forefront of parents’ or clinicians’ priorities. Clinics will need to recommit to or expand their HPV vaccination efforts to ensure adolescents are vaccinated on time.

In circumstances without the added pressure of the pandemic, clinics face challenges implementing existing EBIs to encourage HPV vaccination uptake, such as lack of staff to implement EBIs or QI initiatives, lack of knowledge about which EBIs to implement (9), competing priorities, the need for more staff training, and limited resources (15). With the added stresses of the pandemic, these challenges have been compounded, and many participants reported that because of the need to address pandemic-related issues, HPV vaccination had fallen lower on the list of priorities. This has meant that ongoing QI efforts and EBI implementation to improve vaccination rates were often halted. By necessity, the response to the pandemic has been reactive, rather than proactive, which means that processes that were already in place were not prioritized during the pandemic. To overcome this, clinic staff should refocus on implementing strategies known to work to promote HPV vaccination (eg, reminder/recall, strong provider recommendation) (16) and have been effective in increasing rates for other vaccines (17,18).

Although these interviews focused on challenges presented by the pandemic, several participants spoke about some of their unexpected findings from their efforts to continue providing health care. These participants spoke about how the pandemic was a learning opportunity in keeping patients safe during large-scale outbreaks and noted that they would continue some of their precautions in the future to deal with other infectious diseases. Although the COVID-19 pandemic has had an overwhelmingly negative impact on health care, valuable lessons have been learned about how to continue delivering primary care. However, the time spent to make these changes was at the expense of other ongoing work. For example, many participants spoke about the shift in priorities and the time that was needed to create processes for telehealth visits. Future research should focus on identifying best practices that have been developed during the pandemic to support not only future pandemic responses but potentially also dealing with typical influenza seasons.

Although the topical focus of these interviews was HPV vaccination, results highlight challenges that have likely been present in all implementation work during the pandemic. There have been calls from the implementation science community to use implementation science to address COVID-19 (19,20), but less attention has been paid to how to address the fact that so much of the ongoing implementation work came to a halt during the pandemic. At this juncture, the pandemic is likely far from over, and history shows us that other pandemics and epidemics will occur. Implementation science researchers need to create resilient and sustainable EBIs and implementation processes that are not as vulnerable to emergency situations. This could mean focusing on implementing practices that could be more sustainable in emergency situations, for example, ensuring systems are in place to use reminder/recall messaging. Sustainability has long been a challenge for implementation science, and many implementation studies lack an explicit definition of what sustainability means in practice (21,22), making it even harder for researchers and practitioners alike to focus on best practices in this area. The current situation and data from these interviews highlight that researchers must renew their focus on resiliency and sustainability for EBI implementation.

Our study has several strengths. The primary strength was the use of qualitative methods to gather detailed and descriptive information from a relatively understudied perspective during the COVID-19 pandemic, namely clinic managers working in rural settings. Interviews allowed for detailed and nuanced data from clinic managers about the challenges presented by the pandemic to HPV vaccine delivery and EBI implementation. Additionally, more than three-quarters of participants worked in rural clinics, providing another often-understudied perspective on health care delivery and implementation.

This study also has several limitations, primarily related to the timing of these interviews. When interview recruitment began in August 2020, COVID-19 cases were relatively low in Iowa, but by mid-November cases had risen again; therefore, participants may have had different perspectives on the impact of COVID on their work and organizations. However, despite these limitations, these results offer critical insights into this issue, and future research could focus on understanding perspectives from clinic managers in other geographic areas.

In summary, pre-existing low rates of HPV vaccination coupled with the impact of the pandemic threaten to leave adolescents unprotected against HPV and with increased susceptibility to HPV-related cancers. Our results have short- and long-term implications for both practitioners and researchers working in the fields of adolescent health, HPV vaccination, and implementation science. In the short term, a renewed commitment to EBI implementation for adolescent HPV vaccination is needed to ensure that those who are eligible now as well as those who may have missed doses over the past 2 years are vaccinated. Research conducted before the pandemic found that clinics do not always use EBIs and, when they do, there are significant challenges in implementing them (9,14). These challenges have been exacerbated by the pandemic, and both researchers and clinic staff will need to expend even more effort in this area. For example, for clinics that previously did not have reminder recall systems in place, these systems could be one way to identify all undervaccinated adolescents. However, implementing these new systems may require substantial effort given that the pandemic has taken priority and those involved may need to work even harder to obtain staff buy-in and leadership support. In the long term, the implementation science community needs to create more resilient and sustainable EBIs that can be easily implemented in health care systems. Doing so will help protect future adolescents against missing HPV vaccinations, as well as other preventive health care, during pandemic or emergency situations, like the one we are currently experiencing.

## Post-Test Information

To obtain credit, you should first read the journal article. After reading the article, you should be able to answer the following, related, multiple-choice questions. To complete the questions (with a minimum 75% passing score) and earn continuing medical education (CME) credit, please go to http://www.medscape.org/journal/pcd. Credit cannot be obtained for tests completed on paper, although you may use the worksheet below to keep a record of your answers.

You must be a registered user on http://www.medscape.org. If you are not registered on http://www.medscape.org, please click on the “Register” link on the right hand side of the website.

Only one answer is correct for each question. Once you successfully answer all post-test questions, you will be able to view and/or print your certificate. For questions regarding this activity, contact the accredited provider, CME@medscape.net. For technical assistance, contact CME@medscape.net. American Medical Association’s Physician’s Recognition Award (AMA PRA) credits are accepted in the US as evidence of participation in CME activities. For further information on this award, please go to https://www.ama-assn.org. The AMA has determined that physicians not licensed in the US who participate in this CME activity are eligible for AMA PRA Category 1 Credits™. Through agreements that the AMA has made with agencies in some countries, AMA PRA credit may be acceptable as evidence of participation in CME activities. If you are not licensed in the US, please complete the questions online, print the AMA PRA CME credit certificate, and present it to your national medical association for review.

## Post-Test Questions

### Study Title: Challenges to Adolescent HPV Vaccination and Implementation of Evidence-Based Interventions to Promote Vaccine Uptake During the COVID-19 Pandemic: “HPV Is Probably Not at the Top of Our List”

#### CME Questions

1. Which one of the following statements regarding the application of the human papillomavirus (HPV) vaccine among US adolescents is most accurate?
  - The rate of HPV vaccine completion before the COVID-19 pandemic was approximately 80%
  - The COVID-19 pandemic has been associated with a 75% reduction in the rate of HPV vaccination
  - Providing the HPV vaccine earlier in adolescence has not been associated with greater completion
  - The HPV vaccine is less immunogenic among younger vs older adolescents
2. What were the main domains of challenges to HPV vaccination during the COVID-19 pandemic in the current study?
  - Lack of staff to provide vaccination and lack of supply of HPV vaccines
  - Lack of national guidelines regarding vaccination and worry about the interaction between the HPV and COVID-19 vaccines
  - Both patient and parent resistance to HPV vaccination based on false beliefs
  - Decrease in HPV vaccination in routine care and the negative effect of the pandemic on implementation work
3. All the following variables accounted for patient-related challenges to HPV vaccination in the current study except:
  - Patient fear of contracting COVID-19 in clinics
  - Mandated reductions in in-person clinic visits
  - Increase in telehealth appointments
  - Need to complete in-person sports physical appointments
4. Which one of the following clinic adjustments to the pandemic was cited as an enduring practice change in the current study?
  - Clinic flow to control infection
  - Mandatory vaccine education at each clinic visit
  - Increased use of community-based vaccination
  - Expanded role of reminder systems for vaccination
